# Handgrip Strength and Pulmonary Disease in the Elderly: What is the Link?

**DOI:** 10.14336/AD.2018.1226

**Published:** 2019-10-01

**Authors:** Tatiana Rafaela Lemos Lima, Vívian Pinto Almeida, Arthur Sá Ferreira, Fernando Silva Guimarães, Agnaldo José Lopes

**Affiliations:** ^1^Rehabilitation Sciences Post-Graduate Program, Augusto Motta University Center (UNISUAM), Bonsucesso, 21041-010, Rio de Janeiro, Brazil; ^2^Post-graduate Program in Medical Sciences, School of Medical Sciences, State University of Rio de Janeiro (UERJ), Vila Isabel, 20550-170, Rio de Janeiro, Brazil

**Keywords:** handgrip strength, sarcopenia, pulmonary disease, elderly, rehabilitation

## Abstract

Societies in developed countries are aging at an unprecedented rate. Considering that aging is the most significant risk factor for many chronic lung diseases (CLDs), understanding this process may facilitate the development of new interventionist approaches. Skeletal muscle dysfunction is a serious problem in older adults with CLDs, reducing their quality of life and survival. In this study, we reviewed the possible links between handgrip strength (HGS)—a simple, noninvasive, low-cost measure of muscle function—and CLDs in the elderly. Different mechanisms appear to be involved in this association, including systemic inflammation, chronic hypoxemia, physical inactivity, malnutrition, and corticosteroid use. Respiratory and peripheral myopathy, associated with muscle atrophy and a shift in muscle fiber type, also seem to be major etiological contributors to CLDs. Moreover, sarcopenic obesity, which occurs in older adults with CLDs, impairs common inflammatory pathways that can potentiate each other and further accelerate the functional decline of HGS. Our findings support the concept that the systemic effects of CLDs may be determined by HGS, and HGS is a relevant measurement that should be considered in the clinical assessment of the elderly with CLDs. These reasons make HGS a useful practical tool for indirectly evaluating functional status in the elderly. At present, early muscle reconditioning and optimal nutrition appear to be the most effective approaches to reduce the impact of CLDs and low muscle strength on the quality of life of these individuals. Nonetheless, larger in-depth studies are needed to evaluate the link between HGS and CLDs.

Aging is a physiological, dynamic, and irreversible process that takes place over time. This process occurs gradually, but not uniformly, in cells, tissues, organs, and systems and is associated with a progressive increase in susceptibility to diseases. In the respiratory system, the incidence of chronic lung diseases (CLDs) is comparatively higher in individuals aged 65 and older [1].

The term “sarcopenia” was first introduced to describe the progressive age-related loss of muscle mass and is correlated with poor health-related quality of life (HRQoL) and loss of independence in older adults [2]. Additionally, the term ‘dynapenia’ refers to reduced muscle strength in the elderly. More recently, the European Working Group on Sarcopenia in Older People has recommended the use of indicators of low muscle mass, dynapenia, and poor physical performance to diagnose sarcopenia [3]. In this context, the handgrip dynamometer (HGD) is a useful tool to evaluate muscle strength because it provides simple, fast, reliable, and standardized measurements of total muscle strength. In addition, handgrip strength (HGS) is considered an important measure to diagnose dynapenia because low HGS is a robust predictor of low muscle mass and a clinical marker of poor physical performance [3, 4].

HGS is an indicator of overall physical capacity. It is not limited to assessing the upper limbs and is a good predictor of morbidity and mortality, indicating that the HGD is a potentially useful instrument for evaluating different populations with different respiratory conditions [4-7]. Despite these advantages, HGS is rarely used as a functional measure in patients with respiratory diseases, perhaps because it is erroneously considered a part of a complex battery of functional tests [8].

A growing body of evidence has indicated an association between HGS and poor health outcomes in older people [6, 7, 9]. The age-related decline in skeletal muscle includes the loss of both muscle mass and respiratory muscle strength and thus may impair pulmonary function. The decline in lung function is exacerbated in several respiratory diseases, including chronic obstructive pulmonary disease (COPD), idiopathic pulmonary fibrosis (IPF), and lung cancer (LC). Therefore, the early identification of elderly individuals at high risk of pulmonary function impairment is essential from a public health perspective [10-13]. In this context, the links between HGS and pulmonary function in the elderly have been increasingly investigated. A deeper understanding of the pathophysiology and clinical relevance of the relationship between CLDs and low HGS may facilitate the use of strategies to alleviate the effects of CLDs and improve patient outcomes. Thus, the objective of this narrative review was to analyze the possible links between HGS—a measure of muscle function that is noninvasive, low-cost, and highly reproducible—and CLDs in the elderly.

## MATERIALS AND METHODS

The objectives and search strategy were determined after the formulation of the following research question [14]: “Is there a relationship between hand muscle dysfunction and chronic lung disease in older adults?”

A literature search was performed in Medline, Cochrane Database of Systematic Reviews, Scopus, and Web of Science using the following key words and Boolean operators: (‘elderly’) AND (‘handgrip strength’ OR ‘hand muscle dysfunction’) AND (‘chronic lung disease’ OR ‘chronic obstructive pulmonary disease’ OR ‘asthma’ OR ‘lung cancer’ OR ‘pulmonary fibrosis’ OR ‘usual interstitial pneumonia’ OR ‘idiopathic pulmonary fibrosis’). The inclusion criteria were studies published from the beginning of 2000 until 2018 (observational, clinical trials and reviews), which had investigated HGS, CLDs, and the elderly. Using the reference lists of the retrieved papers, additional articles were identified.

The titles or abstracts were used to exclude duplicate data. If patient data overlapped, we selected the original publication to avoid duplicate data. The full text was reviewed to exclude papers according to the following exclusion criteria: (1) non-English language articles, (2) non-peer-reviewed articles, (3) case series studies, (4) case reports, (5) comments or meeting reports, and (6) editorials. Eligible studies were required to assess at least one of the following clinical conditions: COPD, asthma, LC, and/or IPF.

**Figure 1.**
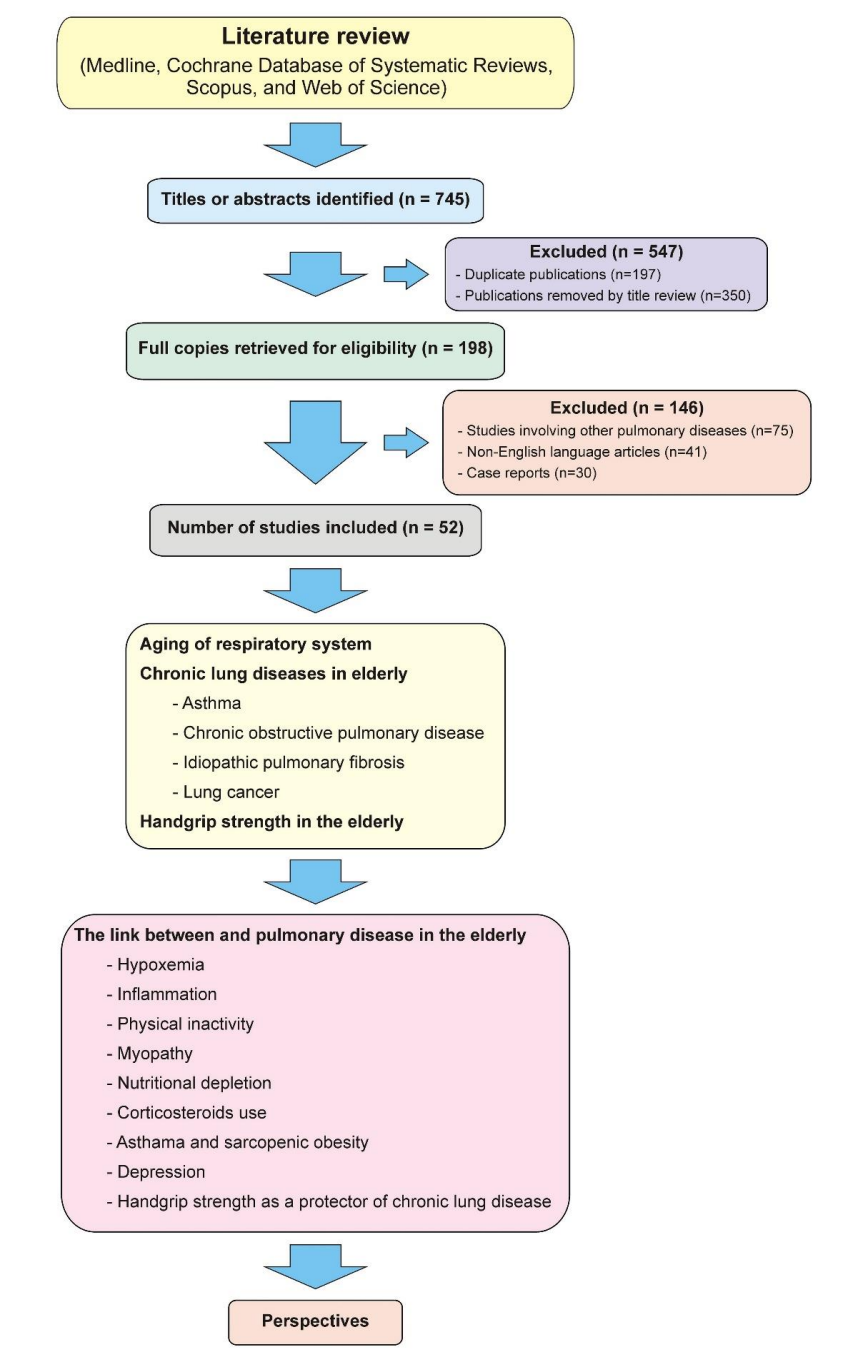
Schematic representation of the scope of the current review.

The analysis was performed by two reviewers, either through the title or the abstract. Subsequently, the reviewers obtained access to the full text of potentially eligible articles, and a detailed analysis was then performed. If there was disagreement between the reviewers regarding the inclusion or exclusion of a study, a third reviewer was asked to evaluate it. Initially, 745 studies were identified. After duplicate removal and eligibility criteria verification, 52 studies were included. The presentation is organized into themes, describing the main research areas in the reviewed literature. A simplified schematic of the study selection flowchart and the main topics discussed is shown in Figure 1.

## RESULTS

### Aging of the respiratory system

The older population is growing faster than younger age groups worldwide. The former is expected to more than double by 2050 and more than triple by 2100, from 962 million individuals in 2017 to 2.1 billion in 2050 and 3.1 billion in 2100 [https://esa.un.org/unpd/wpp/Publications/Files/WPP2017_KeyFindings.pdf]. The increase in the aging population is accompanied by increased health problems, and aging is linked to a decrease in the function of several organs. Skeletal muscle is highly affected by aging, and sarcopenia is the primary manifestation of this process [15]. The prevalence of sarcopenia in the age groups 60-70 years and >80 years is 5-13% and 11-50%, respectively. It is estimated that the total number of cases of sarcopenia to date is at least 50 million individuals and can reach 200 million over the next 40-year period [3, 16].

The lung is one of the few internal organs that are constantly exposed to the external environment; thus, the environmental impact on lung structure and function is a critical determinant of lung health. Prolonged exposure to environmental stressors, coupled with damage to the body's defense systems and genetic predisposition, is more likely to tilt the balance of physiological repair and renewal toward the development of CLDs [17, 18]. In addition, increased breaks in DNA double strands coupled with epigenetic changes, proteostasis loss, mitochondrial dysfunction, cellular senescence, and oxidative stress have been reported in the lung cells of older adults, and these changes may contribute significantly to the increased incidence of disease with increasing age [1, 17].

The natural aging of the lungs is characterized by molecular and cellular changes in multiple lung cell populations. It is believed that the decrease in lung function is due to a reduction in the regenerative capacity of respiratory stem cells [1]. In addition, the number of neutrophils and the release of proteases are increased in the lower respiratory tract of older people, which may contribute to the loss of lung elastic recoil, leading to worsening of pulmonary function [18-21]. With the impairment of the regulatory mechanisms, there is an increase in the risk of fibrosis after lung injury and a reduction in the ability to restore normal lung function. The impairment of regulatory and protective mechanisms progressively reduces the cellular repair capacity and may contribute to the development of age-related CLDs [1, 22].

**Figure 2.**
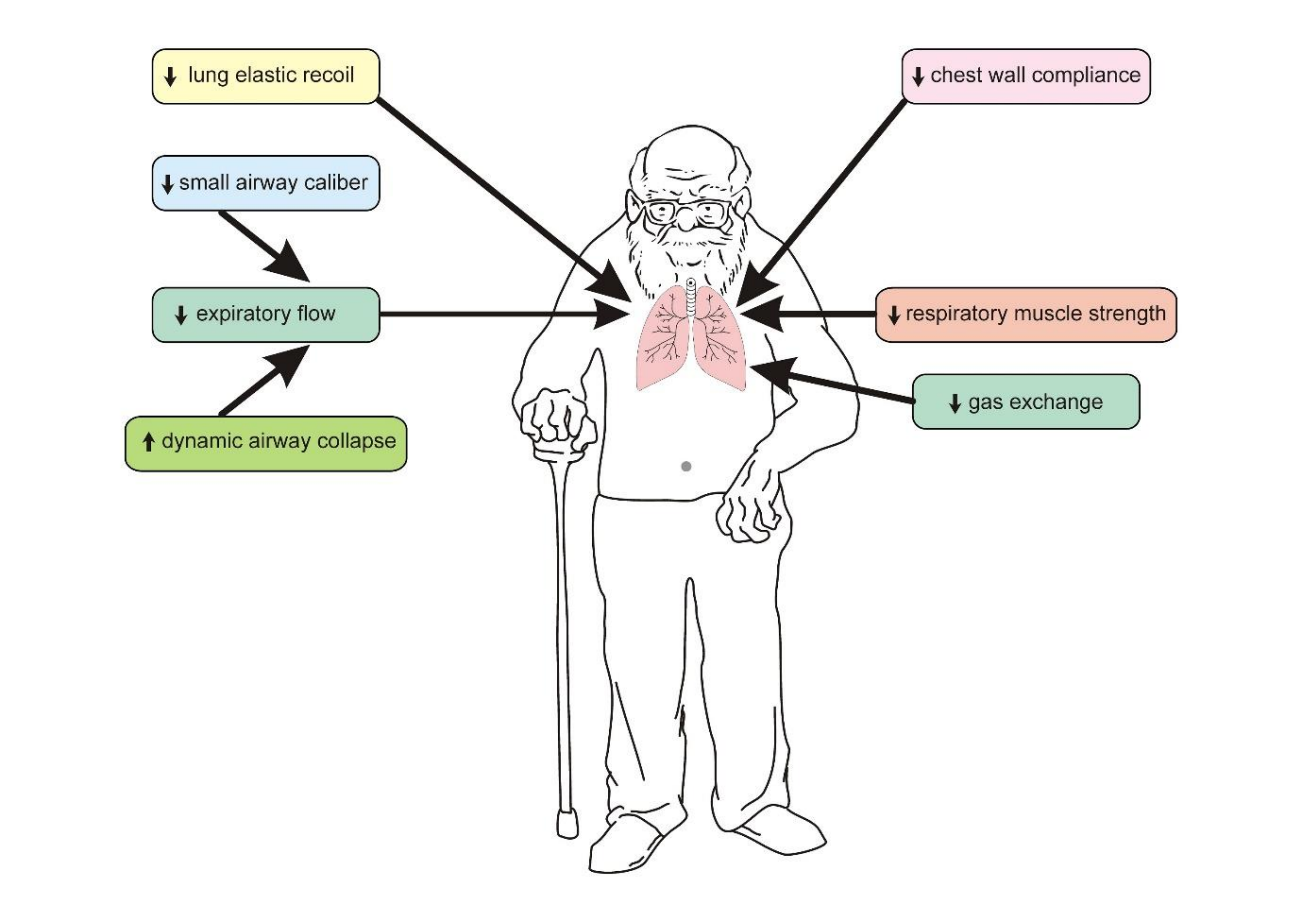
Schematization of the main alterations in the respiratory system of the elderly that may contribute to the pathogenesis of chronic lung diseases.

In the respiratory system, most of the age-related functional changes are due to three physiological events: the progressive reduction in lung elastic recoil, chest wall compliance, and respiratory muscle strength (Fig. 2) [18]. The supporting tissues within the lungs include elastin and collagen, which surround the alveolar ducts and prevent them from collapsing during lung insufflation and deflation [23]. With advancing age, this elastic support network undergoes significant changes, making the lungs less elastic and more compliant. Loss of elasticity reduces lung recoil, which prevents rapid and complete wash-out of the alveolar gas during exhalation [21]. The decline in expiratory flow and air trapping are also dependent on aging, being associated with the progressive reduction of small airway caliber and increased dynamic collapse of the small airways during normal breathing [19]. Other structural changes occur during aging, including an increase in air spaces without destruction of alveolar walls that results in a decrease in the gas exchange surface area and loss of the tissues that support the small airways [18]. Due to the increased collapsibility of the small airways, there is an increase in ventilation-perfusion mismatch with exercise. These characteristics are similar to those of emphysema, except for the absence of deterioration of the alveolar walls [20, 21]. The increase in airspace due to aging is known as ‘senile emphysema’ and can be seen histologically as a fragmentation of elastic fibers in the alveolar septa [21]. In some areas of the lungs, however, the naturally aging lung may also histologically contain increased thickening of alveolar septa, without inflammation or obvious fibrosis [20].

In contrast to the lungs, the aged rib cage becomes stiffer. In fact, the increase in age-related lung compliance is generally counterbalanced by a decrease in chest wall complacency. The stiffening of the rib cage is due to several structural changes, such as calcification of the costal cartilage and joints. In addition, narrow intervertebral discs and age-related osteoporosis may lead to vertebral fractures [21]. With advancing age, there is narrowing of the intervertebral disc spaces, which causes kyphosis or curvature of the spine. This curvature reduces the space between the ribs and creates a smaller thoracic cavity [24]. Moreover, kyphosis and increased sternal convexity lead to structural changes that may result in an increase in the anteroposterior diameter of the thorax and decrease in the curvature of the diaphragm [23]. It is of interest that muscle changes during aging occur concomitantly with changes in lung function, including a reduction in the maximal inspiratory pressure (MIP) and maximal expiratory pressure (MEP) [6]. The reduced respiratory muscle strength is due to mechanical changes in the thorax and intrinsic changes in the muscles themselves. There is atrophy and loss of fast-twitch (type II) muscle fibers, which may predispose the elderly to diaphragmatic fatigue [25].

As a result of anatomical changes characteristic of aging, there is a natural decline in lung function that accounts for much of the morbidity of CLDs [22]. The lungs reach maximum function at approximately 20-25 years of age, which is followed by a steady decline until death. This normal decline occurs even in healthy adults, but it can accelerate due to a variety of respiratory disorders. Decreased lung function is best demonstrated by the nonlinear decline in forced vital capacity (FVC) and forced expiratory volume in 1 second (FEV_1_) [1]. At approximately 30 years of age, FEV_1_ decreases by approximately 25-30 mL per year, and at 70 years of age, this decrease reaches a spectacular rate of 60 mL per year. Importantly, FEV_1_ decreases at a faster rate than FVC, leading to a progressive reduction in the FEV_1_/FVC ratio. Total lung capacity (TLC) remains constant with old age despite the decrease in FVC, whereas functional residual capacity and residual volume increase as a result of increased air trapping [1].

### Chronic lung diseases in older adults

As the population ages, the increase in chronic diseases among patients with decreasing lung function will pose a major public health challenge. In this context, pulmonary diseases have severe consequences in older adults because of age-related changes in respiratory function. Aging is the main risk factor for major noncommunicable CLDs. The incidence of age-related IPF and COPD in individuals aged >65 years increases approximately fivefold, and two-thirds of new cases of LC are diagnosed in this age group [20].

**Figure 3.**
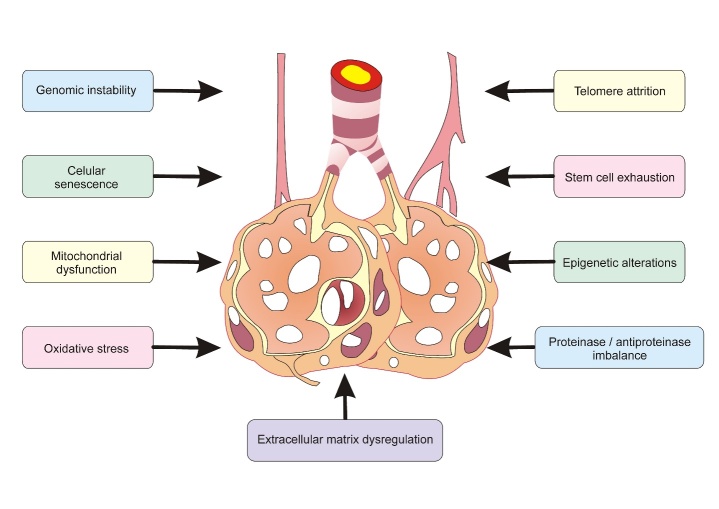
Alterations that cause impairment in the protective mechanisms of the elderly and predisposition to chronic lung diseases.

Pulmonary changes due to aging impair protective mechanisms, which in turn predispose older adults to CLDs (Fig. 3). The mechanisms that lead to genetic changes include genomic instability, telomere attrition, cellular senescence, mitochondrial dysfunction, and oxidative stress, resulting in inadequate cell division [1, 17]. Moreover, stem cell exhaustion, epigenetic changes, and loss of the capacity of the lung tissue to self-repair may be important mechanisms in age-related CLDs [1]. The age-related changes in the delicate balance between extracellular matrix (ECM) proteases and antiproteases significantly increase the susceptibility to COPD, LC, and IPF [1]. Therefore, understanding genetic and epigenetic changes in aging lungs may help to identify age-related risk factors for CLDs.

Current hypotheses corroborate the theory of network medicine, which proposes that diseases are not independent of one another but are a consequence of different mechanisms interacting in a complex network known as a diseasome [26]. In this context, genes involved in distinct respiratory diseases overlap, and these overlaps suggest that similar biopathological processes are the basis for the high variability in clinical cases. There is evidence that CLDs, including IPF, COPD, and LC, are a disease group with overlapping genes. More specifically, the IPF genes *TERT*, *DSP*, and *FAM13A* overlap with LC and COPD genes; with advancing age lung characteristics change, as these genes, which initially had a null effect, augment the risk of disease in aging lungs [20]. Moreover, these three diseases are clinically interrelated by common risk factors, including smoking and epigenetic factors [27].

Although the incidence of COPD worldwide is 200 cases per 10,000 persons in the age group <45 years, the incidence increases to 1,200 per 10,000 persons in the age group >65 years [https://goldcopd.org/]. Considering that age and smoking are the most important risk factors for COPD, the disease burden is likely to increase, especially in countries with aging populations [22, 24, 28]. The age-related incidence of IPF increases almost 5-fold, and the incidence increases to approximately 4-17 cases per 10,000 persons in the age group >75 years [17]. Other studies have provided strong evidence that two important cell types—epithelial cells and fibroblasts—are strongly correlated with aging in IPF [1, 17]. Furthermore, LC and aging are considered two different conditions of the same underlying process, although how age-related changes increase the risk of LC is still not fully understood. It is believed that stems cells with defective DNA, which are common in the elderly, may give rise to tumor cells [1].

In addition to the three clearly age-related diseases (IPF, COPD and LC), it is important to highlight that in older adults, asthma is usually more severe and presents as a phenotype with more severe symptoms and impaired lung function, accompanied at times by weight gain [19, 29, 30]. A study showed that the prevalence of asthma in the elderly increased in the past decade from 6% in 2001 to 8.1% in 2010. Moreover, older adults have the highest rate of asthma-related deaths and the second-highest rate of asthma-related medical consultations and hospitalizations [31]. Additionally, the incidence of difficult-to-control asthma is relatively higher in older adults, probably because of bronchial inflammation, reduced lung function, and adiposity [19]. Of note, the rate of overlap between asthma and COPD (known as asthma-COPD overlap syndrome) is increased in the elderly, and these individuals have worse HRQoL and higher mortality [27].

### Handgrip strength in the elderly

The main role of the skeletal muscle is to perform body movements by generating force. However, aging leads to an annual decrease of 1-2% in muscle mass and muscle strength, and this rate of decline tends to increase further in older age groups [6, 16]. Several epidemiological studies and meta-analyses have shown that muscle dysfunction in older adults is linked to physical disability, decline in activities of daily living (ADLs) and HRQoL, reduced cognitive function, depression, hospitalization, and increased medical costs and mortality, even after adjusting for confounding factors [6, 7, 32]. The estimated heritability of muscle strength in older adults is 40-65%, and a common genetic variant in the chromosomal region that regulates myotube differentiation and muscle repair may contribute to the variability of HGS in older people [33]. Therefore, the elderly should maintain a sufficient level of muscle function to counteract the catabolic effects of aging.

Considering that muscle strength seems to play a critical role in preventing chronic diseases, the measurement of HGS has gained popularity in the past few decades, and it has been incorporated as an important tool in evaluating physical fitness in older adults. HGS is associated with trunk extension strength, elbow flexion strength, and knee extension strength; moreover, it estimates total muscle strength and is considered a robust predictor of mortality and disability [6, 34, 35]. Sillanpää et al [6] evaluated 135 healthy elderly individuals and observed that HGS, but not knee extension torque, was significantly correlated with better pulmonary function as assessed by spirometry, suggesting that age-related mobility decline may be caused by a decrease in muscle strength and muscle power but is also mediated by decreased lung function. Son et al. [36] evaluated 605 older women without chronic diseases and found that HGS was positively associated with lung function in a dose-dependent manner. Given the importance of maintaining adequate lung function, the timely detection of lower HGS may help assess the potential deterioration of lung function in older adults.

**Figure 4.**
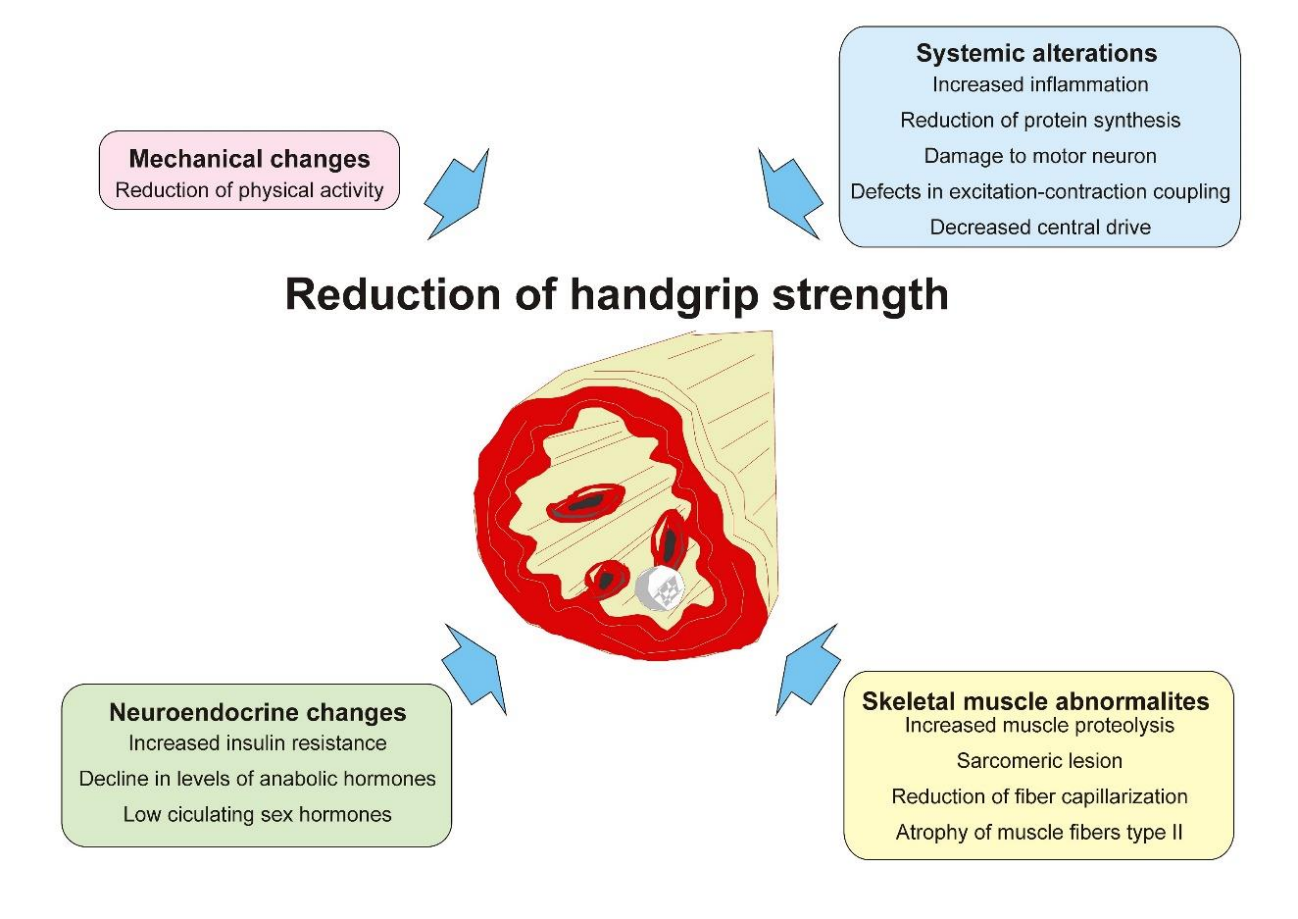
Scheme showing the pathophysiology of age-related changes in muscle tissue.

In healthy older adults, the reduction initiates a cascade of events that contribute to poor lung function and restricted mobility. The pathophysiology of age-related changes in muscle tissues may involve a combination of mechanical, systemic, neuroendocrine, and muscle changes (Fig. 4) [6, 16, 33]. One of the most important mechanical effects is the decrease in physical load with reduced physical activity and training, which is accentuated with advancing age. The decrease in HGS in older people is linked to changes in the type and structure of muscle fibers, excitation-contraction coupling, genetic factors, and oxidative stress. However, some studies suggest that muscle atrophy and the loss of type II muscle fibers and motor neurons, which play an important role in anaerobic metabolism, are the primary mechanism initiating the decline in muscle strength [15, 37]. More recently, Scimeca et al. [38] demonstrated that the activation of the vitamin D receptor, which is one of the most widely studied candidate genes for sarcopenia, is closely associated with the percentage of atrophic muscle fibers.

### Research on chronic lung diseases and handgrip strength in the elderly

Muscle dysfunction is one of the most commonly studied systemic manifestations in CLDs and may be secondary to multiple mechanisms, including dietary changes, systemic inflammation, reduced muscle repair, oxidative stress, drug use, aging, and muscle deconditioning, among others. Muscle dysfunction may appear early in lung impairment, which significantly aggravates the symptoms and directly affects HRQoL [39]. Importantly, patients with CLDs experience a relatively high metabolic load and dyspnea during the performance of ADLs involving the upper limbs compared with healthy controls [40]. The two main mechanisms that explain this finding are the neuromechanical dysfunction (thoracoabdominal asynchrony) of the respiratory muscles and the changes in lung volume during activities involving the upper limbs [40].

#### Chronic obstructive pulmonary disease

Assessing physical performance in patients with COPD is challenging and is still poorly quantified in clinical practice. The prevalence of sarcopenia in these patients varies according to world region and is reported at 14.5% in the United Kingdom and 24% in Thailand [41, 42]. The possible explanations are the variation in the methods used to diagnose sarcopenia and the differences in body composition of individuals of different ethnicities, with Asian individuals seeming to have a higher prevalence of sarcopenia than individuals from other regions [42]. Skeletal muscle dysfunction has been suggested as a cause of poor physical function, similar to the occurrence of sarcopenia and loss of function in healthy aging [10]. However, several studies have shown that loss of muscle mass and muscle strength is more pronounced in older people with COPD than in healthy elderly adults and that COPD progression contributes to loss of muscle mass, functional capacity, and independence [10, 11]. Moreover, systemic COPD symptoms, including dyspnea and general fatigue, may decrease exercise tolerance, favoring a vicious cycle of generalized weakness, sedentarism, and inactivity [11]. Muscle loss does not affect all COPD patients but is more common in patients with an emphysema phenotype than in those with a chronic bronchitis phenotype. It is worth noting that the emphysema phenotype (pink puffer-type of COPD) has long been linked to the loss of muscle mass and body mass [43].

A study evaluating 520 patients with COPD and 150 matched controls found a significant reduction in HGS among these patients (27.1±9.7 vs. 31.3±10.3, *P*<0.001) [10]. These authors observed that HGS was inversely correlated with the results of the timed up and go test (*r*=-0.27, *P*<0.001) and St. George’s Respiratory Questionnaire (*r*=-0.26, *P*<0.001); however, HGS was not associated with the bioinflammatory markers that were studied (C-reactive protein and fibrinogen). Another study reported a significant correlation between HGS and the Medical Research Council (MRC) dyspnea scale (*r*=-0.35, *P*=0.002) and the COPD assessment test score (*r*=-0.24, *P*=0.048) in subjects with COPD [44]. Therefore, HGS may provide relevant prognostic information for patients with COPD. In this respect, Burtin et al [45] have shown that the identification of handgrip weakness provides prognostic data in addition to known predictors such as the ADO (age, dyspnea, airflow obstruction) index and body mass index (BMI) and may play a role in the rapid multidimensional assessment of patients with COPD; furthermore, HGS was strongly linked to mortality in this study. More recently, Gale et al. [46] showed that frailty was predicted by HGS and by the number of exacerbations and comorbidities in a sample consisting of patients with COPD and healthy controls. In that study, the authors discussed several factors that may be involved in the low HGS and frailty of COPD patients, including loss of skeletal muscle mass, loss of bone mineral density, and increased systemic inflammation.

Martinez et al. [8] performed a longitudinal study with 272 subjects with COPD and demonstrated that HGS was related to computed tomography markers of airway thickness and body composition (area of the pectoral muscle and subcutaneous adipose tissue) independent of BMI and emphysema. These authors observed that there was a significant correlation between HGS and FEV_1_ (*r*=0.47) and TLC (*r*=0.54) and that the risk of exacerbation increased 5% for each 1 kg decrease in HGS (risk ratio, 1.04; 95% CI, 1.01-1.07). Similarly, Cortopassi et al. [47] found an association between HGS and COPD severity markers, including the inspiratory capacity (IC) to TLC ratio (*r*=0.77). It is of interest that these authors found that lung hyperinflation (LH) and peripheral muscle performance worsened over one year, whereas the degree of airway obstruction (assessed by FEV_1_) remained relatively unchanged. These findings suggest that functional measures depend on the same factors that explain cardiovascular fitness in COPD, especially LH. Of note, Frohnhofen and Hagen [28] reported that a threshold of 10 kg for HGS allowed reaching the inspiratory flow required for inhalant use in the elderly with COPD and that HGS was the only parameter strongly correlated to inspiratory flow. These authors suggest that HGS should be measured in all older individuals with COPD before making decisions about the inhalant prescription.

**Table 1:** Main findings that support a link between different chronic lung diseases and reduced handgrip strength in the elderly.

Disease	Finding	References
COPD	Loss of pulmonary elastic recoil together with ventilation-perfusion mismatch lead to poor peripheral musculature oxygenation	[10, 23, 24, 47, 57]
	There is an association of respiratory and peripheral muscle weakness with HGS impairment	[10, 11, 13, 25, 28, 36]
	Slow-to-fast twitch fiber type transformation results in increased respiratory work and reduced HGS	[11, 39, 42, 45]
	High sympathetic nervous activity causes increased muscle vasoconstrictor stimulation	[55]
	Endothelial dysfunction and cardiac damage with low oxygen pulse affect muscle function	[59-61]
	Coexistence of peripheral arterial occlusive disease contributes to a lower HGS	[61]
	Low-grade chronic inflammation (inflamm-aging) affects skeletal muscle function	[7, 24, 44, 66]
	Oxidative stress and high IL-6 and TNF-α levels reduce muscle function	[1, 18, 39, 44]
	Muscle atrophy, loss of type I fibers and lower oxidative enzyme activity impair muscle function	[44]
	Reduced muscle fiber capillarization negatively impacts muscle activity	[4]
	Low levels of anabolic hormones may influence the decline in muscle strength	[34, 37, 48]
	Sedentary lifestyle and physical inactivity are related to lower HGS	[8, 10-12, 40, 41, 46]
	Accelerated intracellular protein degradation causes myopathy	[43]
	Malnutrition acts as a contributor to the sarcopenic state and reduced HGS	[77, 78]
	Both systemic and inhaled chronic corticosteroid therapy reduce protein synthesis and increase muscle proteolysis	[4, 42, 81, 82, 92]
	Depression acts as a contributor to the decline in HGS	[24, 34]
	Acute exacerbations of the disease and need for intensive care further reduce HGS	[8, 92]
	There are interrelationships between frailty, worse HRQoL, mortality and low HGS	[34, 40, 46, 53]
Asthma	Loss of pulmonary elastic recoil and increased small airway closure volume cause peripheral muscle hypoxia (especially in acute exacerbations)	[23]
	Persistent, low-grade systemic inflammation causes accelerated muscle catabolism	[19, 67]
	Proinflammatory cytokines cause injury to skeletal muscles	[19]
	Reduction of *insulin-like growth factor I* contributes to muscle dysfunction	[48]
	There are interrelationships between difficult-to-control asthma, long-term asthma and decreased HGS	[67]
	Sex hormones may play a role in explaining intrinsic differences in HGS between genders	[48]
	Corticosteroid use negatively impacts HGS in difficult-to-control asthma	[67, 83]
	The combination of sarcopenia and obesity (sarcopenic obesity) exposes common inflammatory pathways that enhance each other	[16, 54, 86, 87]
	Increased ROS production and secretion of adipokines and cytokines such as IL-6, TNF-α and mcp-1 affect skeletal muscle quality	[16, 87]
	Increased insulin resistance and dysregulation in the hypothalamic-pituitary-adrenal axis increase the risk of depression	[16, 37]
IPF	Oxidative stress and catabolic inflammatory processes increase sarcopenia	[1, 62, 63, 70]
	Mitochondrial dysfunction and accumulation of aged mitochondria damage skeletal muscle	[1, 17, 70]
	The cumulative amount of corticosteroids independently predicts reduced muscle strength	[61, 62, 72, 81]
	Muscle fiber disorganization and cellular metabolic changes impair metabolic demand in episodes of disease exacerbation	[23, 24]
	Physical deconditioning and long-term disease are associated with lower HGS	[73]
Lung cancer	Mitochondrial dysfunction and ROS increase muscle fatigue	[69]
	Increased energy expenditure, changes in metabolism and cachexia compromise peripheral muscles	[51, 79, 80]
	Possible tumor-host interactions may cause muscle dysfunction	[49, 51, 79, 80]
	Gluconeogenic precursors are crucial for maintenance of muscle function and are related to survival	[9]
	The amount of skeletal muscle mass directly impacts survival	[48-51]
	“Acute sarcopenia” secondary to hospitalization aggravates peripheral muscle performance	[74]
	Lifestyle change imposed by cancer worsens HGS reduction	[9, 51]

Note: COPD, chronic obstructive pulmonary disease; IL-6, interleukin 6; TNF-α, tumor necrosis factor-α; HGS, handgrip strength; HRQoL, health-related quality of life; ROS, reactive oxygen species; IPF, idiopathic pulmonary fibrosis

#### Other lung diseases

The association between pulmonary function and HGS has been observed in some cases of CLDs. Cheung et al [48] evaluated 1,154 subjects and found an association between low HGS and chronic airway diseases (including asthma) in men but not in women. These authors hypothesized that low HGS might be used as a marker of subclinical inflammation, including the increase in interleukin (IL)-6 and the decrease in insulin-like growth factor I. In the study, the patterns of association between HGS and chronic airway disease were different between the sexes, which may be due to intrinsic sex differences, including different sex hormones. Celis-Morales et al [9] performed a population-based prospective study with 502,293 participants and demonstrated that higher HGS was linked to a lower risk of all-cause mortality. Moreover, these authors showed that higher HGS was usually related to lower rates of, and mortality due to, respiratory diseases and all cancers, including LC [9].

In patients with LC, the rate of sarcopenia was up to 74% and was associated with decreased survival [49]. The results of a meta-analysis of 38 studies evaluating the predictive ability of sarcopenia for the development of solid cancers indicated that the prognosis of survival increased according to the increase in skeletal muscle mass [50]. In this context, the HGD may be a useful tool to assess the functional and nutritional status of patients with LC. Barata et al [51] evaluated 27 patients with nonresectable LC and observed that HGS was below the 50th percentile in 57% of patients; furthermore, there was a significant relationship between HGS and nutritional status (*P*=0.026, 95% CI). The results of a meta-analysis indicated that cases of solid cancers complicated by low skeletal muscle strength were correlated with poor prognosis (hazard ratio, 1.44; 95% CI, 1.32-1.56) [50].

A previous study demonstrated the reliability of the HGD in measuring muscle strength in interstitial lung disease (including IPF) [52]. Furthermore, HGS was decreased in this population, and a minimal clinically important difference of 1.9 kg and 3.7 kg was necessary to reflect a real change if the tests were conducted by one examiner or two different examiners, respectively.

**Figure 5.**
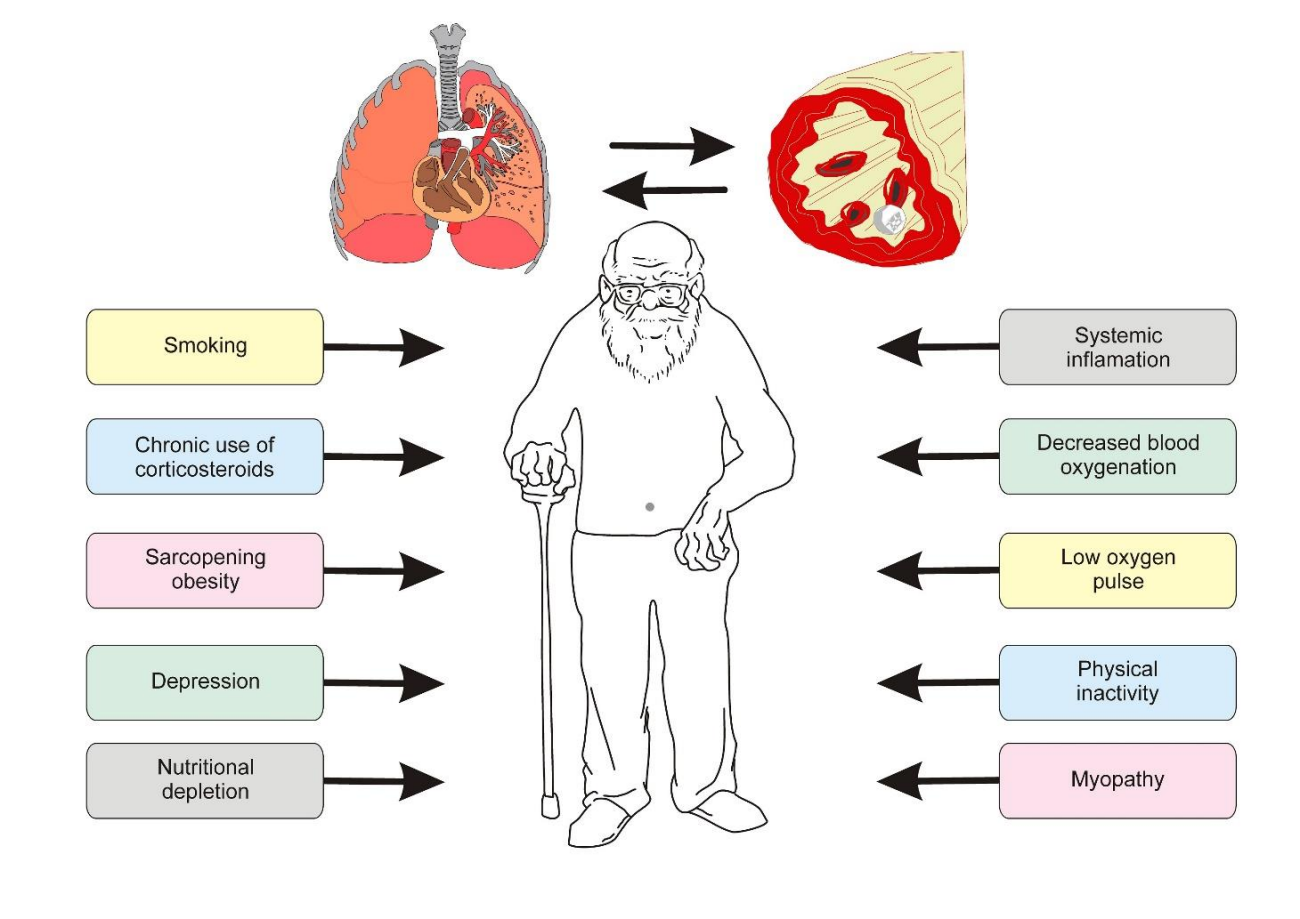
Possible mechanisms of the linkage between handgrip and pulmonary disease in the elderly.

### What is the link?

The concomitant reduction in several functions in different biological systems during aging, including lung function, muscle strength, and muscle power, suggest the presence of common underlying mechanisms in healthy and diseased elderly patients [6]. From the conceptual point of view, the decline in the functional status of elderly individuals with CLDs has been described as a downward spiral of increased disease severity, with increased symptoms and reduced functional capacity and performance [13, 53]. The magnitude of muscle dysfunction is usually correlated with the severity of CLDs, although this correlation is variable, and patients with advanced CLDs may have a relatively preserved muscle integrity [43]. The main findings that support a link between different CLDs and reduced HGS in the geriatric population are shown in Table 1.

Dynapenia is linked to reduced lung function. The fact that HGS strongly predicts functional disability, longevity, and mortality supports the hypothesis that systemic factors such as inflammation, low levels of anabolic hormones, and poor nutritional status, as well as changes in the muscles involved in lung and muscle function, may be more pronounced during the decline in HGS [6, 34-37, 54]. In older adults with CLDs, skeletal muscle weakness can be due to aging, inactivity, smoking, and use of corticosteroids, among other factors [11]. Intrinsic peripheral muscle factors may lead to muscle weakness, including hypoxia, atrophy of type II fibers, and reduced fiber capillarization [4]. Oxidative stress and sarcomeric damage also occur and ultimately decrease the levels of contractile proteins [55]. Therefore, different pathways may be involved in the link between low HGS and CLDs in the elderly (Fig. 5).

#### Structural alterations of the respiratory system and its systemic consequences

In the elderly, the reduction in lung function is caused by several factors related to the pulmonary tissue, including fewer alveoli and capillaries, which limits gas exchange. In addition, an increase in the closing volume of the small airways combined with the stiffening of pulmonary vessels results in a ventilation-perfusion mismatch due to ventilation and perfusion defects [22-24]. In the presence of CLDs, changes in lung volume may exacerbate these defects. The loss of lung elastic recoil is a prominent feature of aging and a characteristic of asthma and COPD regardless of aging [23, 56-58]. CLDs in the elderly cause a significant decrease in lung function and blood oxygenation; these changes may impair the performance of skeletal muscles and result in loss of muscle strength and power [6].

Some mechanisms may explain the close relationship between HGS and pulmonary function in older adults with CLDs. It has been reported that the main parameters representing respiratory muscle strength are correlated with overall muscle strength, indicating that the respiratory and peripheral muscle strength are interrelated. Enright et al [58] performed a large cohort study with older adults and showed that MIP was strongly associated with HGS, suggesting that the effects of aging were similar in both muscle groups. Skeletal muscle mass decreases with age and may lead to dysfunction in respiratory muscles, especially the diaphragm. Respiratory muscle strength plays an essential role in the performance of the respiratory system, which modulates the interaction between lung function and respiratory muscles to maintain adequate ventilation [36].

Although respiratory muscle strength is decreased with advancing age, respiratory muscle performance is also associated with changes in age-dependent chest cavity geometry, reduced chest wall compliance, and decreased lung elasticity. These changes are enhanced in elderly individuals with CLDs and may result in chronic carbon dioxide retention and low blood oxygenation [18]. Therefore, chronic hypoxemia may impair the performance of peripheral muscles and HGS. Furthermore, COPD is associated with higher sympathetic nervous activity, which may contribute to muscle dysfunction by increasing sympathetic vasoconstriction in muscles. In this respect, Haarmann et al. [55] demonstrated that intermittent noninvasive ventilation decreased sympathetic activation during exercise using dynamic HGS and therefore may improve muscle function in COPD patients.

One of the most important systemic effects of LH in COPD is the impairment of cardiac function. Cortopassi et al. [59] evaluated COPD patients and found that HGS was linked to impaired cardiac function measured by the oxygen pulse at rest and during exercise, even when the response was corrected for differences in LH and muscle mass. This result suggests that dysfunctions occur simultaneously in the heart and skeletal muscles in patients with COPD and LH and are not only due to pulmonary mechanics; therefore, low HGS may be a marker of cardiac function impairment in COPD. Accordingly, current studies report that patients with COPD have endothelial dysfunction that may manifest as loss of ability to dilate the brachial artery, probably due to LH and chest wall distension [60, 61]. These changes may negatively impact the HGS. In fact, Miranda et al. [61] recently showed that COPD patients with coexisting peripheral arterial occlusive disease (PAOD) presented lower HGS (33 vs. 26.7 kgf, *P*=0.02) compared with patients with COPD without PAOD.

#### The inflammatory pathway

Conditions such as inflammation or stress may trigger cellular senescence and increase the production of inflammatory mediators and growth factors. Increased senescence has been correlated with pathophysiological mechanisms in most CLDs, including COPD and IPF, in which inflammatory catabolic processes may increase sarcopenia [1, 7, 44, 62, 63]. Systemic inflammation due to CLDs or their comorbidities has been increasingly associated with increased morbidity and mortality [64]. In this respect, a model of interconnected vascular diseases has been proposed, in which the lung is the source of signals that induce inflammatory responses, even in peripheral muscles [65].

**Figure 6.**
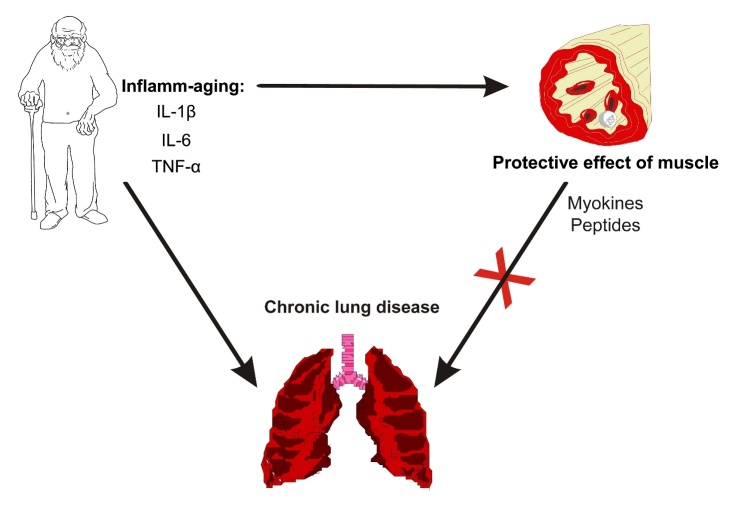
Relationship between inflamm-aging, muscle function, and chronic lung disease in the elderly. While inflamm-aging can lead to damage to muscle and lung tissue, muscle can exert a protective effect on the genesis of chronic lung diseases. IL-1β = interleukin 1β; IL-6 = interleukin 6; TNF-α = tumor necrosis factor-α.

In older adults, innate immunity is impaired, and pulmonary inflammation is exacerbated [17, 18, 34]. The profile of proinflammatory cytokines in this population is characterized by the predominance of IL-1β, IL-6, and tumor necrosis factor-α (TNF-α), which maintains low-grade chronic inflammation, known as inflamm-aging [66]. The increased levels of these proinflammatory cytokines may have local and systemic adverse effects in the elderly. In addition, increased levels of proinflammatory cytokines promote the development and maintenance of immunosenescence, favors the development of CLDs by destroying the pulmonary parenchyma and decreasing lung elasticity, and impairs other organs, including the skeletal muscle (Fig. 6) [19, 24]. Therefore, “inflamm-aging” may be a significant link between the establishment of CLDs and the reduction in HGS.

Systemic inflammation may be an important contributor to sarcopenia in clinically stable populations with COPD. In this respect, Byun et al [44] observed that HGS was negatively correlated with the levels of IL-6 (*r*=-0.27, *P*=0.018) and TNF-α (*r*=-0.35, *P*=0.002). The causes and prevalence of systemic inflammation and the mechanisms underlying muscle dysfunction in COPD are uncertain, although previous studies suggest that severe muscle loss may result in cachexia during systemic inflammation [18, 44]. Moreover, there is a strong negative correlation between IC and IL-6 levels (*r*=-0.781, *P*=0.04), suggesting a relationship between LH and systemic inflammation, which in turn may negatively impact HGS [39]. Stenholm et al. [67] evaluated 963 elderly individuals and reported that asthma in middle age, among other factors, predicted a decline in HGS over a 22-year follow-up period. These authors observed that long-term and low-grade systemic inflammation in asthma might be involved in pathophysiological mechanisms leading to accelerated muscle catabolism.

Exposure to tobacco directly damages type II alveolar cells, which lose their ability to regenerate stem cells, and healthy cells cannot replace damaged cells. In addition, the DNA of functioning endothelial progenitor cells becomes defective, which compromises cell differentiation [1]. A previous study has shown that cigarette smoke also induces changes in mitochondrial structure and function, increasing reactive oxygen species (ROS) and decreasing apoptosis [68]. Another study has shown that smokers have reduced HGS and greater fatigability than nonsmokers [69]. Considering that several lung diseases are caused by smoking, including COPD, IPF, and LC, smoking may be a link between lung diseases and decreased HGS because of the lower regeneration capacity of stem cells.

Oxidative stress in the lungs and other organs plays an important role in the pathogenesis of COPD [18]. Furthermore, the expression and activity of antioxidant enzymes are decreased in COPD, as also occurs during normal aging [18]. The rate of mitochondrial dysfunction is relatively higher in impaired type II alveolar cells. This phenomenon may be related to lower respiratory capacity and increased oxidative stress due to mitochondrial dysfunction. Aging mitochondria accumulate in multiple tissues, including skeletal muscle. For this reason, pathways involved in maintaining mitochondrial integrity have been of particular interest for understanding the link between CLDs (including IPF) and reduced HGS in the elderly [70]. More recently, a study showed that *heme oxygenase-1* attenuates senescence in the fibroblasts of COPD patients and thus protects against mitochondrial dysfunction and restores mitophagy [71].

#### Physical inactivity as a contributor to handgrip strength reduction

Physical inactivity is a predictor of mortality in elderly persons with CLDs and may aggravate sarcopenia [11, 72]. In addition to the disease per se, symptoms such as dyspnea and fatigue and comorbidities maintain the low levels of physical activity in CLD subjects [11]. In this respect, it is well established that less physically active individuals have lower HGS; therefore, the lifestyle changes imposed by CLDs can aggravate the reduced HGS [8, 9, 41]. Dysfunctions tend to be heterogeneous in different muscle groups, which supports the hypothesis that muscle deconditioning due to disuse is an important cause of muscle damage in CLDs [13, 39].

A major effect of COPD is the progressive decrease in physical performance, which may lead to a reduced capacity to perform ADLs [34, 40, 46]. Physical inactivity has detrimental effects on body composition, which may impact the HRQoL and increase systemic inflammation and muscle mass loss [44]. Physical inactivity occurs even when airflow obstruction is mild, as demonstrated by the continuous monitoring of ADLs, in which patients spend less time walking and standing than their matched controls [10]. The consequence is a vicious cycle of physical inactivity and changes in body composition, which decrease physical performance and HGS. The hypothesis of decreased function is supported by the fact that many features of muscle dysfunction in COPD resemble those associated with immobility, including atrophy, loss of type I fibers, and decreased activity of oxidative enzymes [44].

Garcia et al. [11] compared patients with COPD and healthy controls and observed that mobility (60.4±16.9 vs. 71.1±16.38 points), HGS (75.2±14.9 vs. 75.5±15.1% predicted), daily number of steps (4,865.4±2,193.3 vs 6,146.8±2,376.4 steps), and time spent in moderate to vigorous activity (197.3±146.57 vs. 280±168.9 min) were comparatively decreased in the COPD group (*P*<0.05). There was a positive correlation between the daily number of steps (*r*=0.43, *P*=0.01), dyspnea (*r*=0.44, *P*<0.001), and HGS (*r*=0.42, *P*<0.001). These findings suggest that the systemic effects of COPD, including dyspnea and fatigue, may decrease exercise tolerance, leading to a vicious cycle of muscle weakness, sedentarism, and physical inactivity.

Physical inactivity may have important implications in other CLDs. There is an interrelation between difficult-to-control asthma, physical inactivity, and lower HGS. Long-term asthma may also decrease exercise tolerance, physical activity, and muscle performance. These findings have been well documented by Stenholm et al. [67]. Kozu et al. [73] reported that HGS and the degree of dyspnea, as assessed by the MRC scale, were increased in patients with IPF. These authors found that among the factors that might contribute to the higher impairment of muscle strength with the worsening of dyspnea in patients with IPF is muscle deconditioning due to longer disease duration and long-term oral corticosteroid use. It is also worth mentioning that acute exacerbations of CLD can lead to an acute increase in inflammation that, together with reduced physical activity and muscle disuse during hospital stays, can lead to a reduction in muscle mass and function. This condition has been termed “acute sarcopenia”, referring to the acute loss in muscle mass and function associated with hospitalization [74].

#### Myopathy in chronic lung diseases as a link to reduced handgrip strength

Skeletal muscle is the major repository of proteins in the body, and in chronic conditions such as CLDs, this tissue provides gluconeogenic precursors that are crucial for survival as these diseases progress. Moreover, muscle mass and strength are decreased in many CLDs, and therefore, the physiological role played by skeletal muscle is fundamental for maintaining the functional status of these patients [9, 10].

The theory of myopathy is supported by evidence that some aspects of muscle dysfunction are weakly correlated with the level of physical activity in patients with different CLDs and that, even when the levels of physical activity in healthy subjects and subjects with CLDs are matched, muscle integrity is different between these groups [43]. Several biological factors have been implicated in the pathogenesis of muscle dysfunction in older adults with CLDs, including mitochondrial dysfunction, autophagy, changes in muscle composition, and neurodegeneration. Mitochondria are the main energy-producing organelles in cells and consequently play a fundamental role in maintaining muscle function. Moreover, mitochondrial dysfunction due to the systemic effects of CLDs may contribute to sarcopenia, including changes in quality control and activation of cell death. Furthermore, impaired autophagy may result in the accumulation of aged mitochondria and decrease the bioenergetic state of myocytes, which may lead to reduced HGS [37]. More recently, it has been shown that hyperphosphatemia, which is associated with mortality in CLDs, induces senescence in myoblasts through overexpression of integrin linked kinase. As a consequence, the proliferative capacity of myofibroblasts is reduced, leading to the development of sarcopenia and reduction in HGS [75, 76].

Chronic smoking, which is linked to several CLDs, may contribute significantly to the decline in neuronal health in older adults. A major effect is motor neuron degeneration, which is evidenced by a decrease in the conduction velocity and amplitude of maximum muscle action potentials, which may result in decreased muscle strength, including HGS [37].

COPD is usually linked to changes in muscle metabolism and a decrease in mitochondrial enzyme levels, which may lead to decreased muscle strength. In these patients, the supply of oxygen to respiratory and peripheral muscles may be deficient as a result of hypoxemia or reduced blood supply [59]. Therefore, different conditions, including hypoxia, hypercapnia, smoking, malnutrition, and immobilization, may lead to higher intracellular protein degradation. Increased intracellular protein degradation is the hallmark of muscle disease and occurs by two main mechanisms—the ubiquitin-proteasome and lysosomal pathways—that coexist in COPD and operate in a coordinated manner. Furthermore, autophagy and lysosomal pathways are induced in the locomotor muscles of stable patients with COPD, and the degree of autophagy is correlated with the severity of muscle disease and impairment of lung function [43]. Defective muscle stem cells, which are common in muscle injuries, may also contribute to limited muscle repair [43]. Therefore, myopathy in CLDs may be responsible at least in part for the decreased HGS in this population.

#### Nutritional depletion, pulmonary disease, and low handgrip strength

Recent studies have indicated the effectiveness of HGD as a tool for assessing nutritional status [15, 50-53]. In fact, poor nutrition is a major contributor to HGS, and there is a relationship between HGS, waist circumference, and BMI [14]. Riviati et al. [15] evaluated 352 elderly patients and demonstrated that those who were aged >75 years had a 2.3-fold increased risk of low HGS, whereas poor nutrition increased the risk of low HGS by 1.9-fold. Muscle dysfunction in poor nutrition is due to the lower supply of muscle protein, which is an efficient alternative energy source [15, 34]. The low intake of branched-chain amino acids and foods containing antioxidants, vitamins, and carotenoids may result in nutrition-related sarcopenia [72].

Aging is associated with dysregulated inflammatory responses, which may contribute to the pathophysiology of the nutritional status and cause functional decline. During inflammation, IL-6 inhibits the synthesis of albumin and induces the synthesis of C-reactive protein. Therefore, the catabolic effect of IL-6 is believed to promote protein imbalance over time, which may culminate in sarcopenia and low HGS [34].

Poor nutrition is common in patients with COPD or IPF and is related to increased morbidity and mortality. In patients with COPD, nutritional status and body composition, especially when evaluated using fat-free mass (FFM), are related to the presence of skeletal dysfunction and sarcopenia and, more importantly, to the general prognosis [77]. In an evaluation of 263 patients with COPD, Blasio et al. [78] demonstrated that the prevalence of sarcopenia was significantly higher in patients with malnutrition (71.2% vs. 12.3%; *P*<0.001), especially in patients with systemic inflammation (85.7% vs. 61.3%, *P*<0.001). In the same study, the authors also observed that malnourished patients with sarcopenia had a significant reduction in BMI, FFM and HGS compared with non-sarcopenic patients.

In patients with CL, poor nutrition increases the risk of complications, decreases the response and tolerance to treatments, and reduces the HRQoL [50, 79]. This condition is estimated to affect 46% of patients with LC [51]. The etiology of poor nutrition in these patients is multifactorial and involves anorexia, systemic inflammation, metabolic changes, increased energy expenditure, and tumor-host interactions. These factors in combination result in cachexia, sarcopenia, and reduced HGS [51]. Of interest is that the HGD can be used to identify early changes in the nutritional status of patients with LC, because during nutritional deprivation, loss of muscle strength precedes changes in muscle mass and composition [80].

#### The interaction between corticosteroid use and reduced handgrip strength

There is evidence that hormonal dysregulation in the elderly with CLDs affects several physiological systems, including skeletal muscle. Sustained high levels of corticosteroids have been shown to impair muscle function [37]. Long-term corticosteroid therapy induces skeletal muscle weakness, primarily by increasing muscle proteolysis and decreasing protein synthesis [42, 81]. For this reason, lower HGS in patients with CLDs may be in part due to the prolonged use of corticosteroids, both systemic and inhaled [4].

Hanada et al. [62] evaluated 47 patients with lung fibrosis treated with corticosteroids and 51 patients with lung fibrosis not treated with corticosteroids and observed that HGS was significantly lower in the former group (63.8±22.4 vs. 81.8±28.3% predicted, *P*<0.001). In that study, HGS was inversely correlated with the cumulative number of corticosteroids administered (*r*=-0.40, *P*=0.005) and was an independent predictor of lower muscle strength. In COPD, the skeletal muscle may be affected by the prolonged use of corticosteroids, as either oral drugs or inhalants [4]. In addition, patients with COPD and corticosteroid myopathy have a lower survival rate than those not suffering from corticosteroid myopathy [82]. With regard to asthma, Bowyer et al [83] reported that more than 50% of patients receiving >40 mg prednisone daily had evidence of muscle weakness. Similarly, Stenholm et al. [67] observed that corticosteroid use decreased HGS in patients with difficult-to-control asthma. Therefore, the long-term use of corticosteroids may be an important link between CLDs and reduced HGS [62, 67, 73, 82].

#### Asthma, sarcopenic obesity, and handgrip strength

The prevalence of obesity has increased substantially in recent decades, particularly among older adults [84]. Obesity is a multifactorial chronic disease, and its burden is expected to increase in the coming decades concurrently with an increase in older age groups [85]. Although the onset and progression of functional decline are typically associated with sarcopenia, age-related changes in muscle composition appear to be even more pronounced in older adults with obesity. Therefore, muscle dysfunction and obesity may act synergistically to increase the risk of physical disability in obese adults [37, 54].

Both aging and obesity can promote disability, impair organ function, and increase the rate of chronic diseases and inflammation [16]. In this context, a possible link between asthma and HGS may be adiposity. The close association between asthma and obesity in older people is well known, and asthmatic older patients usually have uncontrolled asthma, impaired lung function, and higher BMI [19].

Sarcopenic obesity (SO) is characterized by sarcopenia combined with obesity or increased fat mass. SO is defined by a decrease in appendicular skeletal muscle mass and an increase in BMI, body fat percentage, or waist circumference and is frequently observed in individuals with severe asthma [86]. The combination of sarcopenia with obesity affects common inflammatory pathways that can potentiate each other and may further accelerate functional decline in older people, including HGS decline. In muscle tissues, there is an increase in ROS production and secretion of adipokines and cytokines, including IL-6, TNF-α, and mcp-1, resulting in mitochondrial dysfunction and poor muscle quality control [16, 42]. In addition to inflammation, other factors have been implicated in SO pathogenesis, including fat infiltration in muscle tissue, muscle denervation, weight gain secondary to physical inactivity, and leptin resistance [16]. Interestingly, HGS is lower in patients with SO than in patients with either sarcopenia or obesity [16]. More recently, Xiao et al. [87] observed that SO was a strong univariate predictor of asthma (OR, 2.77, 95% CI, 1.12-6.83). Therefore, asthma combined with SO may predispose people to lower HGS [87].

#### Depression linking lung disease and reduced handgrip strength

There is a positive and bidirectional association between depressive symptoms and functional deficit, and elderly individuals with functional deficits suffer from depressive symptoms more often than healthy older people [37]. Recently, there has been discussion regarding the chronic immune activation that occurs during the aging process, where sarcopenia may be a consequence of the counterregulatory strategy of the immune system. In this scenario, the kynurenine pathway is induced, and elevation in the ratio of kynurenine to tryptophan concentrations may aggravate neuropsychiatric conditions such as depression [88]. Depression is also a potential contributor to HGS decline and increased mortality and is linked to an increased risk of mortality and faster decline in muscle strength. It is estimated that 40% of individuals with COPD suffer from depression, compared to 15% of the general population [24]. Considering that CLDs other than COPD, including IPF and LC, predispose a depressive state, depression may be an important link between CLDs and lower HGS [34].

Chronic inflammation in individuals with CLDs (especially asthma) and SO leads to an increase in insulin resistance and dysregulation of the hypothalamic-pituitary-adrenal axis, elevating the risk of depression. Obesity contributes to cognitive deficits, which in turn increases the risk of disabilities and functional decline in HGS [37]. In addition, the stress resulting from the hypermethylation of glucocorticoid receptors in obesity, especially when associated with asthma, contributes to the development of depression, which in turn is linked to reduced HGS [16].

#### The reverse pathway: handgrip strength as a protector of chronic lung disease

New evidence suggests that skeletal muscle releases several myokines and peptides into the bloodstream in response to muscle contraction [89]. Furthermore, the anti-atherogenic and anti-inflammatory properties of these substances may protect against a number of chronic diseases, including CLDs (Fig. 6) [90]. It has been suggested that muscle strength decline initiates a chain of events leading to lower lung function, poor physical performance, and restricted mobility [6]. Moreover, there is evidence of disorganization of muscle fibers in older adults and, at the cellular level, metabolic changes and a smaller reserve of mitochondrial adenosine triphosphate necessary to sustain a sudden increase in metabolic demand, which may occur in conditions such as exacerbated COPD, asthma attack, and exacerbated IPF [24]. In this respect, older adults with CLDs should maintain a sufficient level of muscle mass and muscle strength to counteract the adverse catabolic and inflammatory effects of aging, especially in situations in which there is an increased risk of respiratory failure.

The prevalence of multimorbidity in the elderly ranges between 58 and 73% and is expected to increase with the increase in life expectancy [7]. In this context, HGS measurement is useful for identifying individuals with muscle weakness who are at high risk of CLDs and who may benefit from further assessments for early detection of CLDs. Notably, Volaklis et al. [7] evaluated 1,079 older adults and found that reduced HGS was correlated with increased multimorbidity (including CLDs) among elderly women, even after adjusting for confounders such as inflammatory markers and level of physical activity. The authors suggest that improving HGS in older women is fundamental to decrease the risk of multiple chronic diseases. Rantanen et al. [34] evaluated 919 older women and showed that HGS was a powerful predictor of respiratory and total mortality during a 5-year follow-up period. However, in contrast to the results of other studies, these authors observed that this association was mediated by mechanisms other than inflammation, poor nutritional status, depression, smoking, or physical inactivity.

In COPD, because of the slow-to-fast twitch fiber type shift (decrease in the number of type 1 fibers relative to the number of type 2 fibers), patients produce more lactic acid and carbon dioxide for a given muscle load, which requires a greater compensatory effort during breathing. This condition may exhaust ventilatory muscle capacity in patients with limited physiological reserves. As a consequence, reduced HGS may adversely affect respiratory system performance in patients with COPD [43]. In a recent retrospective study with a longitudinal follow-up of 60 patients with severe COPD followed for 7 years, Pleguezuelos et al. [91] observed that both HGS and ischiocrural strength (but not quadriceps strength) were independent predictors of mortality. Interestingly, HGS has also been suggested as a good predictor of duration of mechanical ventilation, extubation outcome, and mortality in COPD patients admitted to the intensive care unit [92]*.*

This new approach is of great interest from a public health perspective because muscle strength is a modifiable risk factor and can substantially affect the risk of multiple morbidities, including CLDs. Therefore, improving muscle function, particularly by strength training and rehabilitation for recovery of independence to perform ADL, can improve the HRQoL and significantly reduce mortality by CLDs.

### Perspectives

As the population ages, the increase in CLDs in patients with limited lung function becomes a major challenge, and age-related decline in musculoskeletal function may have a synergistic effect with declining lung function. There are still important gaps in recognizing extrapulmonary functional measures as significant predictors of CLD progression, although body composition and mobility capacity measures have already been included in multidimensional disease impact models such as the BODE (BMI, obstruction, dyspnea, and exercise) index [8]. In this context, HGS may help manage CLDs because this parameter, in comparison with other physical measures such as pulmonary function and physical activity, is easily measurable, low-cost, and highly reproducible [9, 93]. Despite the high rate and prognostic value of low HGS, this functional marker of aging has not yet been included in current guidelines for the evaluation, management, and prognosis of subjects with CLDs such as COPD and IPF [8].

The hypothesized relationship between LH and systemic inflammation should be validated with further research, because this relationship can at least partially explain the reduced HGS in CLDs [39]. Elucidating this relationship is necessary because LH can be assessed in the follow-up of therapeutic interventions for treating muscle dysfunction in patients with CLDs. Therefore, future studies are necessary to assess muscle strength in these patients and determine predisposing factors for muscle dysfunction, considering the presence of LH as an outcome variable. Another factor that needs to be better understood is the interrelation between LH, cardiac damage, and low HGS. LH may compromise ventricular filling in some CLDs, such as COPD and asthma; how this event is related to HGS is still a matter of debate, but the strong relationship suggests more than a random event [47]. Therefore, the close relationship between HGS, LH, and cardiac function found in some studies is intriguing and deserves more attention, because its synchronous change over time suggests a possible common mechanism [47, 59].

Inflammation appears to be one of the strongest links between reduced HGS and CLDs. Therefore, many clinical and basic studies investigating antioxidant and anti-inflammatory agents in the fight against inflamm-aging are being conducted with the view to attenuate the prolonged and exaggerated inflammatory response in the elderly. Further studies on direct and indirect pathways for therapeutic manipulation of inflammation may contribute to the overall health of older adults, possibly improving lung and muscle health and directly impacting HGS [19, 24, 65].

Pulmonary rehabilitation programs (PRP) can improve dyspnea, functional balance, muscle strength, and exercise tolerance in patients with CLDs. In addition, aerobic physical training may help increase skeletal muscle mass and improve motor function, restoring to some extent the normal muscle phenotype [43]. Thus, there is a growing interest in assessing upper limb exercise capacity in patients with CLDs enrolled in PRP to better document the impact of upper limb training [40]. In this context, the HGD may be a useful tool to longitudinally evaluate muscle strength recovery and help monitor the therapeutic efficacy. Combined interventions, including exercise-based treatments (resistance training) and nutritional interventions, are effective in improving sarcopenia and may be recommended [32]. In addition, exercise is effective in increasing skeletal muscle mass and function in patients with LC. In turn, amino acid supplementation seems to increase body weight and improve physical and cognitive functions in patients with COPD. The use of dietary protein supplementation may further increase protein anabolism but may also contribute to a more active lifestyle in the elderly [88]. However, the reason why these approaches improve long-term outcomes is still not fully understood [88, 94].

CLDs in the elderly cannot be adequately managed without considering the complexity of the interaction with comorbidities. This assumption implies a multidimensional analysis to identify priorities and strategies for treating CLDs in older adults. Considering that the treatment of COPD and asthma is based on the use of inhalants, arthritis in the hands and fingers may cause HGS problems and affect the ability to use inhalation devices properly; this, in turn, affects the pharmacological treatment of these conditions [27].

### Conclusions

The association between low HGS and CLDs in the elderly is multifactorial. In fact, current evidence indicates the presence of different phenomena linking lower muscle mass and function with the occurrence of CLDs in this population. Chronic systemic inflammation is related to nontransmissible CLDs in the elderly, and this inflammatory status may be one of the main links to reduced HGS. In addition to systemic inflammation, other contributors that appear to be important are the chronic effects of hypoxemia due to CLDs, physical inactivity, respiratory and peripheral myopathy, malnutrition, and the use of corticosteroids, which is common in many CLDs. SO is increasingly diagnosed in different clinical conditions and may be an important link between decreased HGS and adiposity in CLDs. Reduced HGS in CLDs should be considered a systemic phenomenon requiring a holistic approach to restore physical reconditioning and nutritional status. Therefore, early targeted interventions should be developed in patients with CLDs to delay muscle strength decline and prevent functional limitations and disabilities. Furthermore, longitudinal, randomized, and controlled studies are needed to identify the correlation between muscle strength, pulmonary function, and functionality in elderly people with CLDs as well as the impact of rehabilitation programs on those outcomes.
